# Therapeutic Notes

**Published:** 1898-03-05

**Authors:** 


### THERAPEUTIC NOTES.

### The Bath Treatment of Phagedæna.

Among the more dangerous complications of chancroid ulceration is the occurrence of phagedæna, either in the original sore or in the groin. It is usual to attempt to arrest this process by the application of strong caustics, such as nitrate of mercury, fuming nitric acid, or the actual cautery. Mr. Swinford Edwards strongly advises, as a far better and simpler treatment, continuous immersion in hot water. The patient sits in his bath upon a circular india-rubber cushion; over the top of the bath a blanket is placed, through which a hole has been made to admit his head, and he stays there four hours at a stretch, an attendant being at hand to draw off the water as it cools and to supply fresh hot water.

